# Glutathione Peroxidase-1 Primes Pro-Inflammatory Cytokine Production after LPS Challenge *In Vivo*

**DOI:** 10.1371/journal.pone.0033172

**Published:** 2012-03-06

**Authors:** Steven Bozinovski, Huei Jiunn Seow, Peter J. Crack, Gary P. Anderson, Ross Vlahos

**Affiliations:** Department of Pharmacology, The University of Melbourne, Victoria, Australia; Hannover School of Medicine, Germany

## Abstract

Reactive oxygen species produced during the innate immune response to LPS are important agents of anti-pathogen defence but may also cause oxidative lung damage. Glutathione peroxidase-1 (gpx-1) is an anti-oxidant enzyme that may protect lungs from such damage. We assessed the *in vivo* importance of gpx-1 in LPS-induced lung inflammation. Male wild-type (WT) or gpx-1 deficient (gpx-1^−/−^) mice were treated intranasally with PBS or 10 µg LPS and killed 3 and 24 h post LPS. Lungs were lavaged with PBS and then harvested for inflammatory marker expression. LPS caused an intense neutrophilia in WT BALF evident 3 and 24 h post challenge that was reduced in gpx-1^−/−^ mice. In addition, LPS-treated gpx-1^−/−^ mice had significantly fewer macrophages than LPS-treated WT mice. To understand the basis for this paradoxical reduction we assessed inflammatory cytokines and proteases at protein and transcript levels. MMP-9 expression and net gelatinase activity in BALF of gpx-1^−/−^ mice treated with LPS for 3 and 24 h was no different to that found in LPS-treated WT mice. BALF from LPS-treated gpx-1^−/−^ mice (3 h) had less TNF-α, MIP-2 and GM-CSF protein than LPS-treated WT mice. In contrast, LPS-induced increases in TNF-α, MIP-2 and GM-CSF mRNA expression in WT mice were similar to those observed in gpx-1^−/−^ mice. These attenuated protein levels were unexpectedly not mirrored by reduced mRNA transcripts but were associated with increased 20S proteasome expression. Thus, these data suggest that gpx-1 primes pro-inflammatory cytokine production after LPS challenge *in vivo.*

## Introduction

The innate immune response is critical for host defence by detecting and removing invading pathogens. Inhaled airborne pathogens are normally sensed by pathogen recognition receptors predominantly expressed on the respiratory epithelium and macrophages which in turn coordinate host defence mediated by proteolytic, cellular and oxidative stress mechanisms [1]. LPS is an endotoxin from the outer membrane of gram-negative organisms and a primary trigger of innate immunity and acute inflammation, which are essential for successful antimicrobial defence reactions against such organisms [1]. In many cell types, LPS induces inflammation by binding to soluble LPS-binding protein, which then facilitates binding to membrane-associated accessory proteins, particularly membrane CD14 (mCD14) and MD-2 and at least one signalling-competent co-receptor where mammalian Toll-like receptor-4 (TLR-4) has been implicated [1]. LPS triggers activation of myeloid differentiation protein (MyD88), MyD88-associated protein Mal, TNF-receptor-associated factor-6 (TRAF-6), IL-1-receptor-associated kinase (IRAK), NF-κB, and Akt [1]. These signal transduction intermediary molecules in turn up-regulate and promote the production of inflammatory cytokines such as TNFα, IL-1β, neutrophil-recruiting chemokines, oxygen radicals and proteases which are part of the inflammatory defence reaction [1].

Inhaled LPS is an important cause of environmentally induced airway disease in occupations where exposure to bacteria-contaminated organic dusts (bioaerosols) is common [2]. In human volunteers and in several animal species, lung aerosol challenge or LPS instillation causes a neutrophil-rich inflammatory response [3], [4], [5]. LPS is released from pathogens such as *Moxarella cataraliss*, *Haemophilius influenzii* and *Pseudomonas aeruginosa*, which are known to acutely infect and also to colonize the lungs of patients suffering from asthma, COPD and cystic fibrosis [6], [7], [8]. LPS from these organisms has therefore been implicated in worsening airway inflammation and non-viral exacerbations of these conditions [4], [6]. In addition, environmental exposure to LPS-containing bio-aerosols was identified as actually causing as well as exacerbating asthma [6], [9], [10]. Therefore, understanding the regulation of inflammation triggered by delivering LPS into lungs is likely to be relevant to both enhancing host defense in gram-negative lung infections and dampening detrimental inflammation in some chronic lung diseases or their exacerbations.

Reactive oxygen species (ROS) are a family of highly reactive molecules that are produced by a variety of cell types in the lung in response to chemical and physical agents in the environment [11]. It is well known that ROS are critical in host defence as they kill invading pathogens. However, their excessive accumulation, or their impaired clearance, in the lung results in oxidative damage including DNA damage, lipid peroxidation and protein denaturation [11]. The cell has developed enzymatic defences to combat excessive production of ROS, including glutathione peroxidase (gpx).

Glutathione peroxidases are a family of selenium-dependent and independent antioxidant enzymes which catalyze the reduction of damaging hydrogen peroxide (H_2_O_2_) as well as a large variety of hydroperoxides (such as DNA peroxides and lipid peroxides) into water and alcohols, respectively, and thus protect bio-membranes and cellular components against oxidative stress [12]. Analysis of the selenoproteome has identified six gpx isoforms in mammals. The cytosolic selenium-dependent gpx-1 is the predominant isoform of cellular gpx and is ubiquitously expressed throughout the body [12]. Sources in the lungs include epithelium, alveolar epithelial lining fluid and alveolar macrophages [13]. We have previously shown that mice lacking the gpx-1 gene are highly susceptible to oxidative-stress and have proposed that gpx-1 may be an attractive target for increasing the anti-oxidant capacity in ischemia/reperfusion brain injury [14], [15], [16] and cigarette smoke-induced lung inflammation [17].

Given that the known biology of gpx-1 appears to be protection during oxidative stress we proposed that gpx-1 protects against LPS-induced lung inflammation. Paradoxically, gpx-1 deficient mice exposed to LPS had significantly reduced levels of BALF macrophages, neutrophils, TNF-α, MIP-2 and GM-CSF protein. In contrast, LPS-induced increases in TNF-α, MIP-2 and GM-CSF mRNA expression in WT mice were similar to those observed in gpx-1^−/−^ mice. Thus, these attenuated protein levels were unexpectedly not mirrored by reduced mRNA transcripts but were associated with increased 20S proteasome expression. Whole lung MMP-9 mRNA, MMP-9 expression, and net gelatinase activity in BALF of gpx-1^−/−^ mice treated with LPS for 3 and 24 h was no different to that found in LPS-treated WT mice. Our data provide new evidence for the role of gpx-1 in LPS-induced lung inflammation and suggest that gpx-1 primes pro-inflammatory cytokine production after LPS challenge *in vivo.*

## Results

### LPS-induced BALF inflammation is reduced in gpx1^−/−^ mice

**Figure 1:**
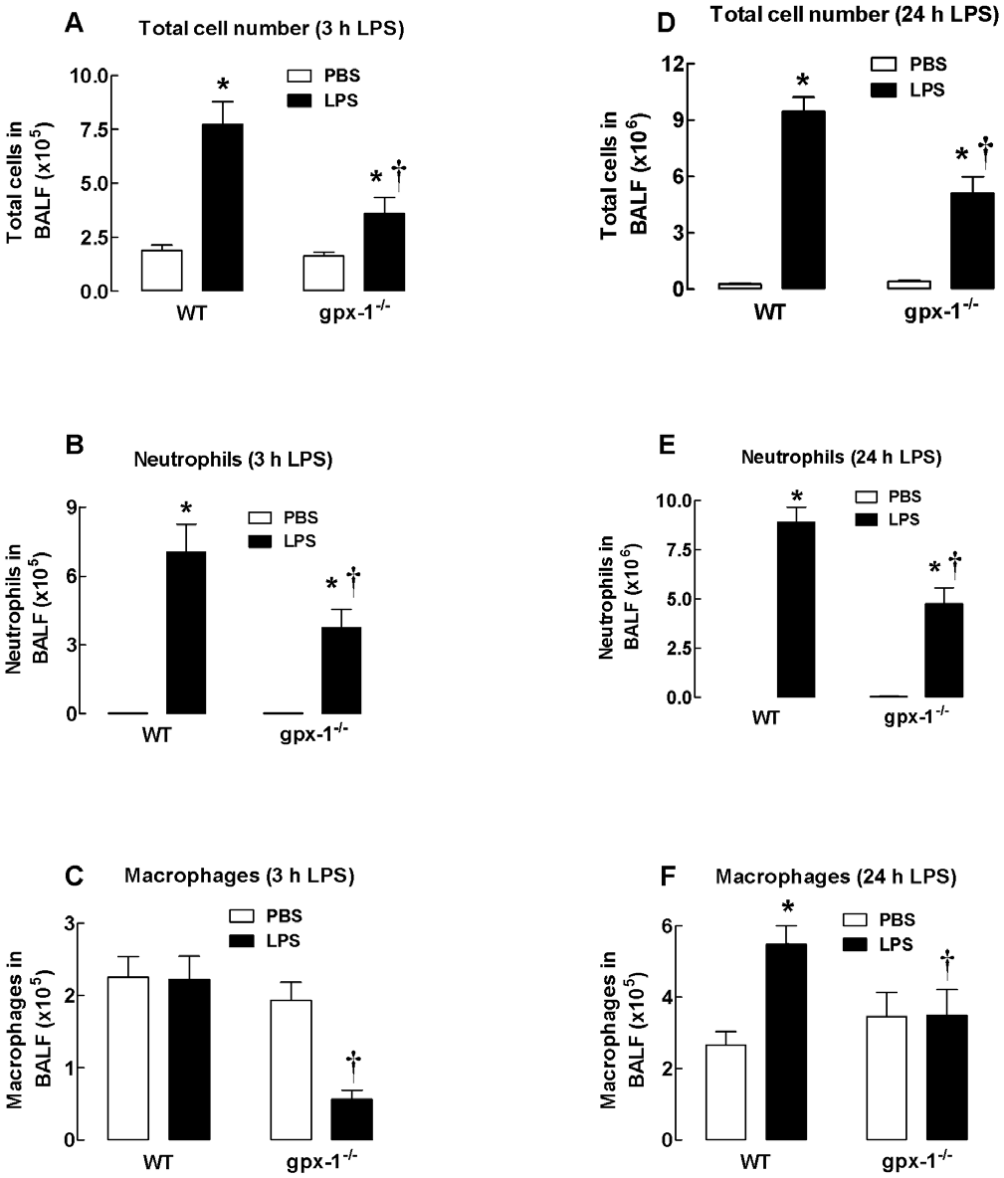
Effect of LPS on BALF cellularity in WT and gpx-1^−/−^ mice. Mice were treated with PBS or LPS (10 µg/mouse) for 3 h (Panels A–C) and 24 h (Panels D–E) and the number of total cells, neutrophils and macrophages counted. Data are shown as mean ± SEM for 6 mice per treatment group. Clear bars represent PBS-treated mice and black bars represent LPS-treated mice. *P<0.001 vs PBS (ANOVA and Bonferroni's *post hoc* test), ^†^ *P*<0.001 vs WT (for total cells and neutrophils, ANOVA followed by Bonferroni's *post hoc* test), ^†^ *P*<0.05 vs WT (for macrophages, ANOVA followed by Bonferroni's *post hoc* test).

WT mice treated with 10 µg LPS for 3 h had significantly more BALF cellularity compared to mice treated with PBS (*P*<0.001, Figure 1A–C). Differential cell count analysis determined that there was a significant increase in the number of neutrophils but no change in macrophage cell number (*P*<0.001, Figure 1A–C). However, gpx-1^−/−^ mice had significantly fewer numbers of total cells, macrophages and neutrophils (*P*<0.001). Total cells, macrophages and neutrophil numbers in gpx-1^−/−^ mice treated with PBS were similar to those in WT mice treated with PBS (Figure 1A–C).

WT mice treated with 10 µg LPS for 24 h had significantly more BALF cellularity compared to mice treated with PBS (*P*<0.001, Figure 1D–F). Differential cell count analysis determined that there was a marked increase in the number of neutrophils and macrophages. However, gpx-1^−/−^ had significantly fewer numbers of total cells, macrophages and neutrophils (*P*<0.001). Total cells, macrophages and neutrophil numbers in gpx-1^−/−^ mice treated with PBS were similar to those in WT mice treated with PBS.

### LPS-induced increases in MMP-9 expression and net gelatinase activity are not affected by gpx-1 deletion

**Figure 2:**
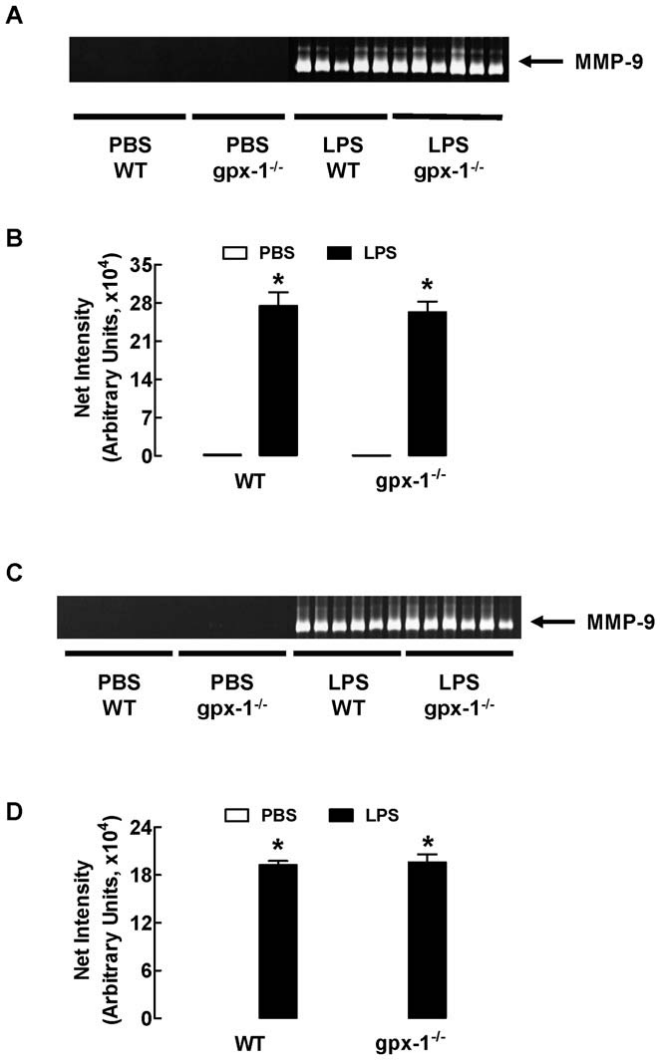
Effect of LPS treatment on MMP-9 expression in BALF of WT and gpx-1^−/−^ mice. WT and gpx-1^−/−^ mice were treated with LPS (10 µg/mouse) for 3 h (Panel A) and 24 h (Panel C) and MMP-9 expression in BALF of individual mice measured by zymogarphy. Panels A & C show MMP-9 assayed by gelatin zymography under reducing conditions and Panels B & D are the respective densitometric measurements. Densitometry data are shown as mean ± SEM for 5–6 mice per treatment group. Clear bars represent PBS-treated mice and black bars represent LPS-treated mice. *P<0.05 vs respective PBS-treated mice (ANOVA and Bonferroni's *post hoc* test).

**Figure 3:**
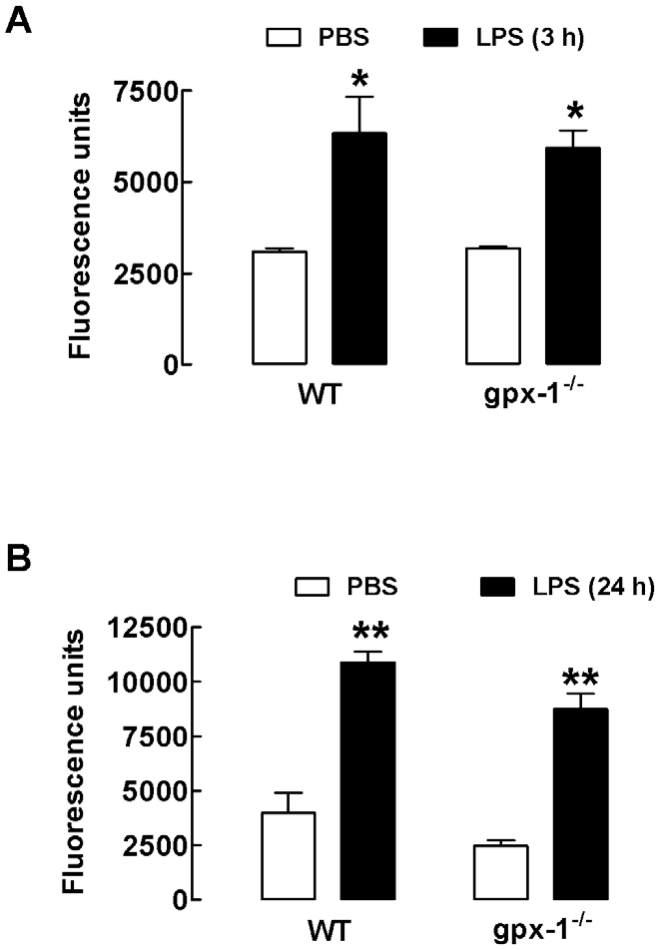
LPS-induced increases in net gelatinase activity is not affected by gpx-1 deletion. Panels A & B show net free gelatinase activity in neat BALF from individual mice treated with LPS for 3 and 24 h, respectively. Data are shown as mean ± SEM for 6 mice per treatment group. Clear bars represent PBS-treated mice and black bars represent LPS-treated mice. *P<0.05 vs respective PBS-treated mice (ANOVA and Bonferroni's *post hoc* test), **P<0.01 vs respective PBS-treated mice (ANOVA and Bonferroni's *post hoc* test).

Since MMPs contribute to the movement of neutrophils and macrophages into the lung parenchyma and MMP-9 expression in response to LPS is transcriptionally regulated by NFκB and AP-1, we measured the secretion of MMP-9 in the BALF of LPS-treated mice by gelatin zymography. There was a marked increase in MMP-9 expression in BALF from WT mice treated with LPS for 3 and 24 h (Figure 2). Consistent with the zymography, there was an increase in net gelatinase activity in the BALF from mice treated with LPS for 3 and 24 h (Figure 3). However, gpx-1^−/−^ mice treated with LPS for 3 and 24 h had similar levels of MMP-9 and net gelatinase activity to LPS-treated WT mice (Figures 2 and 3).

### LPS-induced increases in BALF TNF-α, MIP-2 and GM-CSF protein are reduced in gpx-1^−/−^ mice

**Figure 4:**
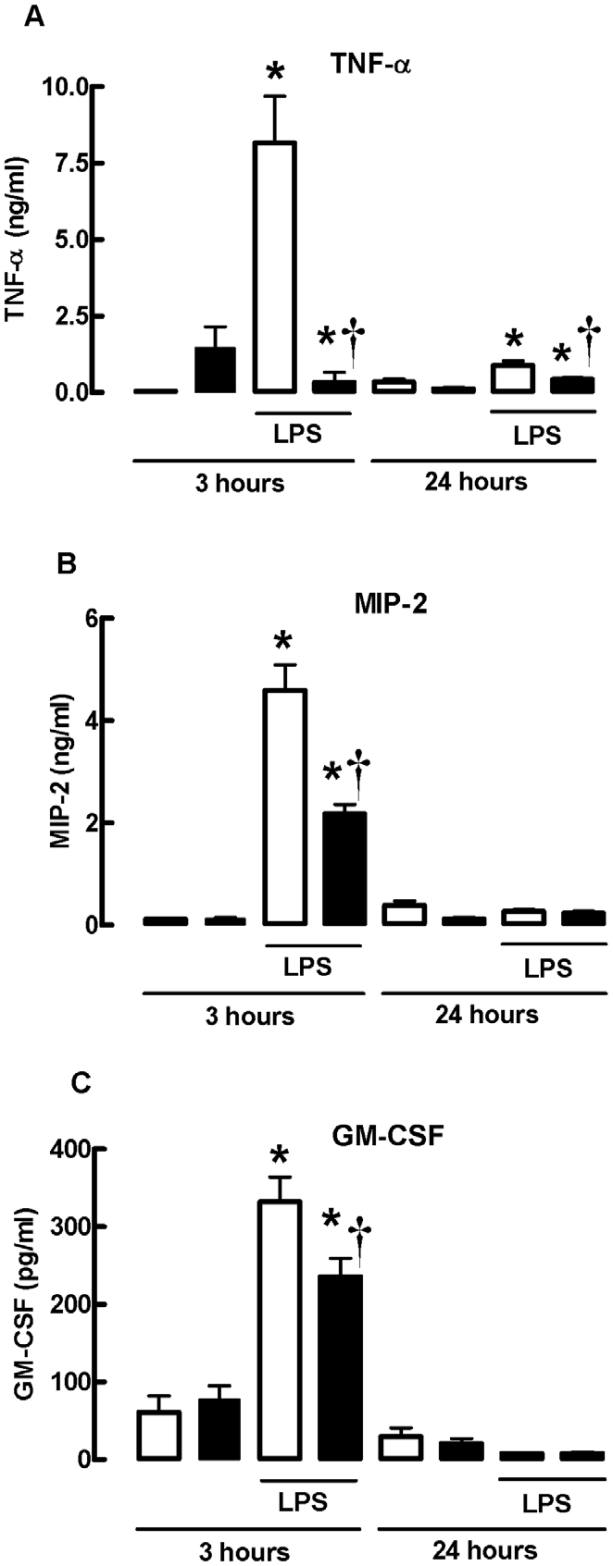
LPS-induced increases in BALF TNF-α, MIP-2 and GM-CSF protein are reduced in gpx-1^−/−^ mice. Data are shown as mean ± SEM for 6 mice per treatment group. Clear bars represent PBS-treated mice and black bars represent LPS-treated mice. *P<0.001 vs respective PBS-treated mice (ANOVA and Bonferroni's *post hoc* test), ^†^ *P*<0.05 vs WT LPS-treated mice (ANOVA followed by Bonferroni's *post hoc* test).

The levels of pro-inflammatory cytokine (TNF-α), chemokine (MIP-2), and leukocyte survival factor (GM-CSF) protein in response to LPS was investigated in WT and gpx-1^−/−^ mice treated with LPS for 3 and 24 h (Figure 4). WT mice treated with LPS for 3 h had significantly more TNF-α, MIP-2 and GM-CSF protein as measured by ELISA in BALF compared to PBS-treated mice (*P*<0.001) (Figure 4). However, gpx-1^−/−^ mice had significantly reduced levels of TNF-α, MIP-2 and GM-CSF protein (*P*<0.05). The levels of TNF-α, GM-CSF and MIP-2 in BALF of LPS-treated WT and gpx-1^−/−^ mice were markedly lower at the 24 h time-point, and no differences in MIP-2 and GM-CSF could be detected. However, LPS-treated gpx-1^−/−^ mice had significantly less TNF-α than LPS-treated WT mice (*P*<0.05).

### LPS-induced increases in whole lung TNF-α, MIP-2, GM-CSF and MMP-9 mRNA are not reduced in gpx1^−/−^ mice

**Figure 5:**
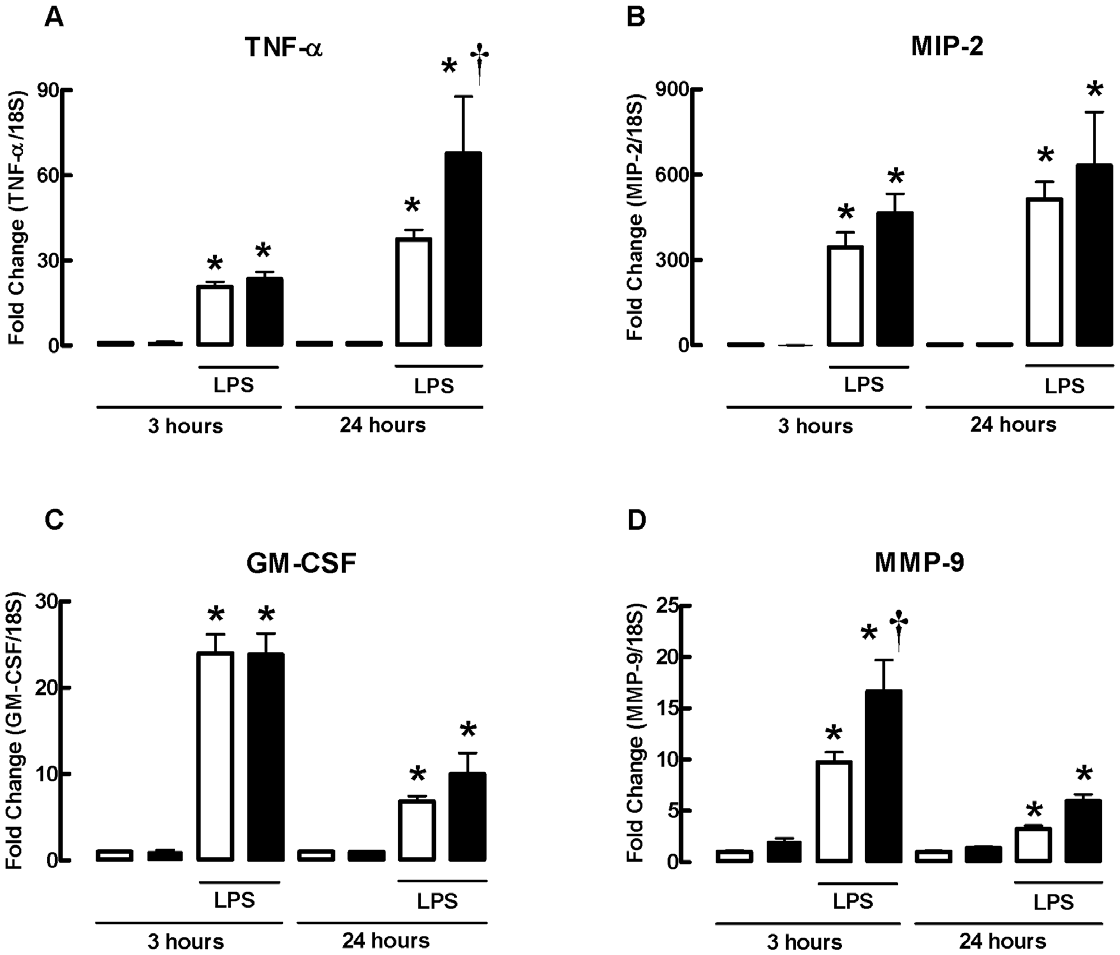
Effect of LPS on whole lung TNF-α, MIP-2, GM-CSF and MMP-9 mRNA expression in WT and gpx-1^−/−^ mice. mRNA expression for all genes was measured simultaneously under identical conditions using quantitative real-time PCR. Responses are shown as fold change relative to 18S from 6 individual mice. Clear bars represent WT mice and black bars represent gpx-1^−/−^ mice. *P<0.001 vs PBS (ANOVA and Bonferroni's *post hoc* test), ^†^ *P*<0.05 vs WT (ANOVA followed by Bonferroni's *post hoc* test).

The expression of TNF-α, MIP-2, GM-CSF and MMP-9 mRNA in response to LPS was investigated in WT and gpx-1^−/−^ mice treated with LPS for 3 and 24 h (Figure 5). LPS treatment of WT mice caused a significant increase in mRNAs for TNF-α, MIP-2, GM-CSF and MMP-9 (*P*<0.001) at both time-points. Gpx-1 deletion did not affect LPS-induced increases in MIP-2 and GM-CSF but enhanced MMP-9 and TNF-α mRNA expression at 3 h and 24 h, respectively.

### The ubiquitin-proteasome pathway is increased in LPS-treated gpx-1^−/−^ mice

**Figure 6:**
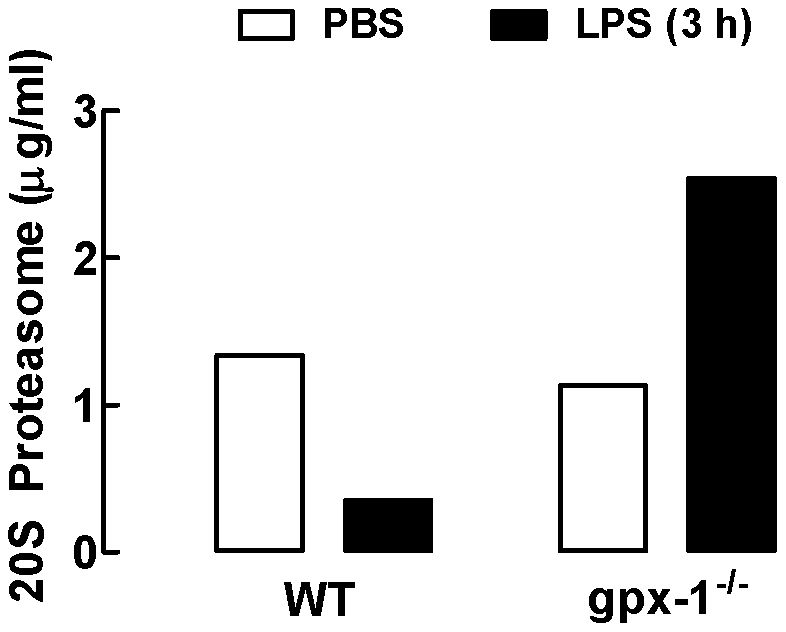
Effect of LPS on 20S proteasome concentrations in BALF of WT and gpx-1^−/−^ mice. WT and gpx-1^−/−^ mice were treated with LPS (10 µg/mouse) for 3 h and 20S proteasome concentrations measured in BALF. Data are shown as the mean for BALF pooled from 5–6 individual mice per treatment group. Clear bars represent PBS-treated mice and black bars represent LPS-treated mice.

The ubiquitin-proteasome system is the major non-lysosomal system for the degradation of short half-life proteins that are involved in basic cellular processes, such as cell-cycle regulation and apoptosis, transcriptional regulation or antigen processing. The proteasome is a multicatalytic proteinase complex that is involved in the selective degradation of proteins. The 20S proteasome is the proteolytic core particle of a large protein degradation complex, the 26S proteasome. In order to explore whether the reduction in BALF TNF-α, MIP-2 and GM-CSF protein observed in LPS-treated gpx-1^−/−^ mice at 3 hours was associated with the ubiquitin-proteasome pathway we measured BALF 20S proteasome concentrations (Figure 6). LPS treatment of WT mice caused a decrease in 20S proteasome levels compared to PBS-treated WT mice. In contrast, LPS-treated gpx-1^−/−^ mice displayed a 2-fold increase in 20S proteasome levels compared to PBS-treated gpx-1^−/−^ mice. Levels of 20S proteasome in PBS-treated gpx-1^−/−^ mice were similar to those of PBS-treated WT mice.

## Discussion

The principle objective of this study was to investigate the role of gpx-1 in a mouse model of LPS-induced lung inflammation. Given that the known biology of gpx-1 appears to be protection during oxidative stress-induced lung inflammation [14], [15], [16], [17] we were surprised to find that gpx-1^−/−^ mice exposed to LPS have reduced BALF inflammation, suggesting that gpx-1 exacerbates LPS-induced lung inflammation *in vivo*.

To understand the basis for the paradoxical reduction of LPS-induced lung inflammation in gpx-1 deficient mice we assessed whether key cytokines and chemokines involved in the recruitment of inflammatory cells in response to LPS were affected. We have previously shown that macrophages and neutrophils are increased in response to LPS in mice [18], [19] which is consistent with other lung LPS-exposure models [3], [4], [20]. LPS can increase cell numbers by promoting cell proliferation, recruitment from the circulation or prolonged survival in the airways. The observed numbers of BALF macrophages increased by LPS exposure in mice has been postulated as representatives of both the progeny of resident alveolar macrophages and influx of blood monocytes adopting macrophage-like morphology (“alveolar monocytes”) [21]. We have previously published that LPS-treated mice had an increased percentage of BAL macrophages in mitosis compared with PBS-treated mice based on nuclear mitotic figures indicating active cell division [18]. Moreover, the increase in BALF neutrophils in response to LPS is in accordance with whole lung gene up-regulation of the chemotactic factor MIP-2. The blood growth factor GM-CSF (a cytokine produced readily by the respiratory epithelium which regulates the activation and survival of macrophages and potently increases neutrophil survival and activation) was also up-regulated. It was interesting to note that the reduced infiltration of macrophages and neutrophils in BALF of gpx-1^−/−^ mice is in accordance with a reduction in BALF concentrations of the pro-inflammatory cytokine TNF-α, the chemotactic factor MIP-2 and the macrophage survival factor GM-CSF. In addition, gpx-1^−/−^ mice treated with LPS for 24 h had markedly less MCP-1 (major monocyte chemotactic factor) mRNA expression than LPS-treated WT mice (90±5 fold change relative to 18S and 247±51, respectively). What was surprising was that these attenuated protein levels were unexpectedly not mirrored by reduced mRNA transcripts in LPS-treated gpx-1^−/−^ mice. However, from our study it appears that the reduction in BALF TNF-α, MIP-2 and GM-CSF protein observed in LPS-treated gpx-1^−/−^ mice at 3 hours was due to protein degradation via the ubiquitin-proteosome pathway. This is consistent with gpx-1 being able to downregulate 20S proteasome activity [22].

TNF-α is considered to be a key inflammatory mediator in lung pathology, and its levels are elevated in lung diseases where LPS is implicated and neutrophilic inflammation is prominent. TNF-α is released by activated alveolar macrophages in response to LPS and is a potent chemoattractant for neutrophils. In concert with chemokines, TNF-α also promotes release of reactive oxygen species, elastase, and other proteases including MMPs that are implicated in lung tissue damage. In mice, neutralization of TNF-α was previously shown to blunt instilled LPS-induced lung inflammation [23]. LPS triggers preformed TNF-α release from mast cells, which is consistent with the very rapid increase in TNF-α levels in our study, and such TNF-α is a potent stimulus for secondary induction of chemokines that in turn promote leukocyte influx and survival. We previously investigated the molecular pathway(s) that mediates this reinforcement of TNF-α levels *in vivo* and in particular demonstrated the GM-CSF-dependent activation of the transcription factors AP-1 and NFκB in response to LPS [19].

GM-CSF has pro-inflammatory properties because of its action on neutrophils and cells of the monocyte macrophage lineage [24] and directly contributes in NFκB-dependent lung inflammation in part via activation of the upstream kinase Akt/PKB [19]. Recombinant GM-CSF augments leukocyte leukotriene and superoxide anion production [25], enhances allergic sensitization [26], and when selectively over-expressed as a transgenic product in the rat lung epithelium, GM-CSF promotes inflammation and fibrosis [27]. In our study, we observed that GM-CSF mRNA was unaltered in gpx-1^−/−^ mice treated with LPS compared with LPS-treated wild type mice. Interestingly, GM-CSF protein was significantly reduced in gpx-1^−/−^ mice treated with LPS. However, given that GM-CSF protein in BALF was not completely abolished, and because GM-CSF inhibits neutrophil apoptosis [24], the reduced neutrophilic inflammation was probably due in part to apoptosis. GM-CSF levels are markedly elevated in lung macrophages and airway epithelium by exposure to LPS *in vitro* [28] and GM-CSF acts directly as a survival factor for neutrophils and macrophages [24]. Some of the associated decline in BALF macrophage numbers could be due to a similar apoptotic cell death: we have previously observed GM-CSF sensitive macrophage replication that is consistent with the ability of GM-CSF to induce lung macrophage replication *in vitro* [29] and the increase in macrophage numbers observed after targeted GM-CSF over-expression in the lung [27]. In addition, we have previously shown that GM-CSF regulates lung innate immunity to LPS through Akt/Erk activation of NFκB and AP-1 *in vivo* [19], and that these responses to LPS are suppressed and reversed by neutralization of GM-CSF via repression of TLR-4 [18].

Proteases which break down connective tissue components are released by multiple cell types in the lung, particularly epithelium and macrophages and, among other functions, contribute to inflammatory cell infiltration into inflamed lungs [30]. MMP-9 induction was previously associated with the development of emphysema in lungs exposed to LPS [31], [32]. In patients with emphysema there is an increase in BALF concentrations and macrophage expression of MMP-9 [33]. Neutrophils can also secrete MMP-9 which can contribute to tissue destruction [30]. In the present study, LPS increased MMP-9 protein expression and net gelatinase activity in BALF of WT mice, correlating to the higher numbers of macrophages and neutrophils observed. It was a surprise that gpx-1^−/−^ mice exposed to LPS had similar BALF MMP-9 protein expression and net gelatinase activity given the reduced numbers of macrophages and neutrophils. In our previous study we found that MMP-9 protein expression was increased in gpx-1^−/−^ mice during cerebral ischemia-reperfusion injury [34]. However, in the present study we found that MMP-9 transcript was higher in gpx-1^−/−^ mice exposed to LPS compared to WT LPS-treated mice. These findings suggest that gpx-1 does not have a role in the control of LPS-induced lung inflammation directed by the protease MMP-9 in mice.

Reactive oxygen species including superoxide, H_2_O_2_, and hydroxyl radical are generated during normal and disease-related metabolic processes and play an important role in modulating cell activation and function [11]. Most studies have assumed that increased generation of ROS result in cellular and organ dysfunction, however recent data suggest that different ROS may have distinct effects on cellular activation. For example, superoxide appears to increase the release of pro-inflammatory cytokines by neutrophils, whereas elevated intracellular concentrations of H_2_O_2_ diminish LPS-induced neutrophil activation and the severity of acute lung injury [12], [35], [36], [37]. Therefore, therapies that increase intracellular concentrations of H_2_O_2_ might be beneficial in neutrophil-driven pro-inflammatory conditions, such as acute lung injury. While we have not measured the levels of intracellular H_2_O_2_ in this study, we would expect that gpx-1 deficient mice have enhanced levels of H_2_O_2_ as the role of gpx-1 is to catalyse H_2_O_2_ into H_2_O and O_2_. Thus, these increased levels of H_2_O_2_ may contribute to the reduced lung inflammation observed in our study.

In summary, the marked suppression of LPS-induced lung inflammation by gpx-1 deletion that we have described, suggests that gpx-1 is a key mediator of LPS-induced lung inflammation. These data suggest that gpx-1 primes pro-inflammatory cytokine production after LPS challenge *in vivo* and suggest the possible therapeutic utility of blocking gpx-1 in human lung diseases where high neutrophil and macrophage numbers, protease induction, and TNF-α, MIP-2 and GM-CSF overproduction are believed to be central agents in disease pathogenesis. However, strategies that promote gpx-1 activity should be used with caution and fully evaluated before clinical acceptance.

## Materials and Methods

### Animal ethics statement

The experiments described in this manuscript were approved by the Animal Experimentation Ethics Committee of The University of Melbourne (AEC Application Numbers 05097, 0810912) and conducted in compliance with the guidelines of the National Health and Medical Research Council (NHMRC) of Australia.

### Animals

Specific pathogen-free male wild type (C57BL/6, 7–10 weeks) mice were obtained from the Animal Resource Centre Pty. Ltd. (Perth, Australia) and gpx-1^−/−^ mice (7–10 weeks, C57BL/6 background) were bred at the Department of Pharmacology, The University of Melbourne. Mice were housed at 20°C on a 12-h day/night cycle in sterile micro-isolators and fed a standard sterile diet of Purina mouse chow with water allowed *ad libitum*.

### LPS administration and bronchoalveolar lavage

Mice were anaesthetized by methoxyflurane (Medical Developments International Ltd, Springvale, Victoria, Australia) inhalation and 50 µl of (a) PBS and (b) maximally tolerated dose of LPS (10 µg of Escherichia Coli serotype 026:B6: Sigma in 50 µl of PBS) administered intranasally as previously described [18], [19]. 3 and 24 h after LPS or PBS treatment mice were killed by an i.p. overdose of anaesthetic (5.6 mg ketamine/1.12 mg xylazine, Parnell Laboratories, NSW, Australia) and the lungs lavaged with PBS as previously published [17], [18], [19], [38], [39], [40]. The total number of viable cells in the BALF was determined, cytospins prepared and cells differentiated by standard morphological criteria. Whole lungs were cleared of blood via right ventricular perfusion of the heart with 5 ml of PBS, rapidly excised *en bloc*, snap-frozen in liquid nitrogen and stored at −80°C until required.

### Protease expression and activity in BALF

Zymography was used to identify the predominant MMP in BALF based on molecular weight and substrate specificity for gelatine as previously described [17], [19], [38], [39]. This approach identified MMP-9 as the predominant gelatine degrading proteinase in our model system. To further quantify gelatinase activity in BALF, we utilised a fluorogenic substrate (fluorescence conjugated gelatine, Life Technologies, Foster City, California, USA) as previously published [17], [19], [38], [39] that only detects free MMP-9 that is not complexed to its endogenous inhibitors (eg TIMPs).

### RNA extraction and Quantitative Real-Time PCR

Total RNA was extracted from 15 mg of whole lung tissue from 6 individual mice per treatment group using RNeasy kits (Qiagen, Valencia, California, USA), reverse transcription with SuperScript III (Applied Biosystems, Foster City, California, USA) and triplicate real time PCR reactions with Applied Biosystems pre-developed assay reagents and 18S rRNA internal control were done as previously described [17], [18], [19], [38], [39], [40].

### ELISAs

Murine TNF-α, MIP-2 and GM-CSF levels were analyzed according to the manufacturer's instructions (R&D Systems, Minneapolis, Minnesota, USA). In addition, 20S proteosome concentrations in BALF were measured with a Proteasome ELISA kit as per the manufacturer's instructions (Enzo Life Sciences, Ann Arbor, Michigan, USA).

### Statistical analyses

As data were normally distributed, they are presented as grouped data expressed as mean±standard error of the mean (s.e.m.); *n* represents the number of mice. Differences were determined by two-way analysis of variance (ANOVA) followed by Bonferroni *post hoc* test for multiple comparisons, where appropriate. All statistical analyses were performed using GraphPad Prism™ for Windows (Version 5.02). Probability levels less than 0.05 (*P*<0.05) were taken to indicate statistical significance.
